# Morroniside improves AngII-induced cardiac fibroblast proliferation, migration, and extracellular matrix deposition by blocking p38/JNK signaling pathway through the downregulation of KLF5

**DOI:** 10.1007/s00210-024-03039-1

**Published:** 2024-03-12

**Authors:** Haotian Zheng, Linxin Yang, Huashang Huang, Yazhou Lin, Lin Chen

**Affiliations:** 1 The Shengli Clinical Medical College, Fujian Medical University, Fuzhou, Fujian 350001 People’s Republic of China; 2 Department of Cardiology, Fujian Provincial Hospital, No. 134 East Street, Fuzhou, Fujian 350001 People’s Republic of China; 3 Department of Ultraphonic Medicine, Fujian Provincial Hospital, Fuzhou, Fujian 350001 People’s Republic of China

**Keywords:** Myocardial fibrosis, Morroniside, p38/JNK signal pathway, KLF5

## Abstract

Myocardial fibrosis (MF), which is an inevitable pathological manifestation of many cardiovascular diseases in the terminal stage, often contributes to severe cardiac dysfunction and sudden death. Morroniside (MOR) is the main active component of *Cornus officinalis* with a variety of biological activities. This study was designed to explore the efficacy of MOR in MF and to investigate its pharmacological mechanism. The viability of MOR-treated human cardiac fibroblast (HCF) cells with or without Angiotensin II (AngII) induction was assessed with Cell Counting Kit-8 (CCK-8). The migration of AngII-induced HCF cells was appraised with a transwell assay. Gelatin zymography analysis was adopted to evaluate the activities of MMP2 and MMP9, while immunofluorescence assay was applied for the estimation of Collagen I and Collagen III. By means of western blot, the expressions of migration-, fibrosis-, and p38/c-Jun N-terminal kinase (JNK) signal pathway-related proteins were resolved. The transfection efficacy of oe-Kruppel-like factor 5 (KLF5) was examined with reverse transcription-quantitative PCR (RT-qPCR) and western blot. In this study, it was found that MOR treatment inhibited AngII-induced hyperproliferation, migration, and fibrosis of HCF cells, accompanied with decreased activities of matrix metalloproteinase 2 (MMP2), matrix metalloproteinase 9 (MMP9), connective tissue growth factor (CTGF), Fibronectin, and α-SMA, which were all reversed by KLF5 overexpression. Collectively, MOR exerted protective effects on MF by blocking p38/JNK signal pathway through the downregulation of KLF5.

## Introduction

As is known to all, cardiovascular disease (CVD) is a predominant contributor to death worldwide (Murtha et al. 2017). Myocardial fibrosis (MF), which is a common histological feature associated with myocardial injury, has been widely reported to be a prognostic factor of adverse cardiac outcomes in different cardiac pathologies (Weber et al. 2013; Bing et al. 2019; Aquaro et al. 2020). It is believed that MF often results from myocardial infarction, coronary, and hypertensive heart disease, as well as aortic stenosis (Zabihollahy et al. 2020).

*Cornus officinalis* is a traditional Chinese herbal medicine in East Asia, and morroniside (MOR) is the most abundant iridoid glycoside in *C. officinalis* (Sun et al. 2020). It has been evidenced that MOR possesses a wide range of biological activities, such as antioxidant, antiapoptotic, anti-fibrosis, and anti-inflammatory effects (Zhang et al. 2017a; Gao et al. 2020; Park et al. 2009). The role of MOR has been extensively discussed in many fibrosis-related diseases because of its anti-fibrotic effect (An et al. 2022). For example, MOR treatment can reduce the expressions of fibrosis marker TGF-β1, α-SMA, and Collagen I in mice with fibrosis (Chen et al. 2022). Besides, An and co-workers have put forward that MOR exerts anti-liver fibrosis effects in vitro (An et al. 2022). Additionally, MOR has been evidenced to exert cardioprotective effects on rats suffering from acute myocardial infarction (Yu and Wang 2018). However, the role of MOR in MF has not been elucidated so far.

As a zinc-finger transcriptional factor, Kruppel-like factor 5 (KLF5) is involved in the development of many diseases, especially in cancers and cardiovascular diseases (Luo and Chen 2021). It has been reported that KLF5 can regulate a variety of cellular processes, including cell proliferation, apoptosis, migration as well as differentiation (Ma et al. 2020). KLF5-specific inhibitor ML264 can decrease the expressions of fibrosis-related markers in myocardial infarction (Zabihollahy et al. 2020). Besides, Xiao et al. have elaborated that p38/JNK pathway is involved in heart failure, and the inhibition of p38/JNK can alleviate cardiac fibrosis (Xiao et al. 2023). Moreover, the downregulation of KLF5 by miR-195 can block JNK signaling pathway (Chang et al. 2020). Interestingly, the Super-PRED database (https://prediction.charite.de/subpages/target_prediction.php) predicts that MOR can target and regulate KLF5.

To sum up, this study was implemented to explore the effects of MOR on the proliferation, migration, and fibrosis in MF as well as to investigate the hidden reaction mechanism, hoping to shed novel insights into the pharmacological treatment for MF.

## Material and methods

### Cell culture and treatment

Human cardiac fibroblast (HCF) cells that supplied from BeNa Culture Collection (Henan, China) were incubated in Dulbecco’s modified Eagle’s medium (DMEM; Gibco) containing 10% fetal bovine serum (FBS; GE Healthcare Life Sciences) and 1% penicillin–streptomycin at 37 °C with the presence of 5% CO_2_. When cell confluence reached 50–60%, HCF cells were used for follow-up experiments. After serum starvation for 12 h, HCF were pre-treated with different concentrations of MOR (5, 10, and 20 µM) for 24 h (An et al. 2022) and then induced by 100 nM Angiotensin II (AngII) for 48 h (Chen et al. 2021). The cells were divided into Control, AngII, AngII + 5 µM MOR, AngII + 10 µM MOR, and AngII + 50 µM MOR groups.

### Cell transfection

pc-DNA3.1 vectors containing the complete sequence of KLF5 (ov-KLF5) and the empty vector (ov-NC) were constructed by GenePharma (Shanghai, China). With the application of Lipofectamine® 2000 reagent (Thermo Fisher Scientific, Inc.), 100 nM recombinants were transfected into HCF cells at 37 °C for 48 h. After 48 h, the cells were collected for follow-up experiments.

### Cell Counting Kit-8 (CCK-8)

HCF cells were injected into 96-well plates at a density of 3 × 10^4^ cells/well and then incubated for 24 h. Subsequently, 10 µL of CCK-8 reagent was added into each well, and the cells were incubated for another 2 h at 37 °C. Finally, the absorbance was detected using a microplate reader (Thermo Fisher Scientific, Inc.) at 450 nm.

### Transwell

HCF cells were injected into 24-well plates at a density of 5 × 10^4^ cells/well and then incubated for 24 h. Following the wash with PBS, HCF cells were injected into the serum-free medium (200 µl) in the upper chambers pre-coated with Matrigel at 37 °C for 1 h, while DMEM containing 10% FBS was inoculated on the lower chambers. After incubating for 24 at 37 °C, the invading cells on the lower surface were fixed with 4% paraformaldehyde and stained with 0.1% crystal violet for 30 min. Finally, the images of HCF cells were observed under an inverted microscope.

### Gelatin zymography analysis

For the detection of MMP2 and MMP9, gelatin zymography protease assay was employed. Briefly, the sodium dodecyl sulfate (SDS) sample was used for the preparation of the collected media with an appropriate volume, and then the media were subjected to 0.1% gelatin-7% SDS-polyacrylamide gel electrophoresis (PAGE). Subsequently, gels were washed with 2.5% Triton X-100 and maintained in reaction buffer for 12 h at 37 °C (Dong et al. 2020). Finally, Coomassie Brilliant Blue R-250 was applied for staining.

### Western blot

Total proteins extracted from sample HCF cells using radioimmunoprecipitation assay (RIPA) lysis buffer (Solarbio) were quantified with bicinchoninic acid (BCA) protein assay kits (Thermo Fisher Scientific Inc.) according to the manufacturer’s instructions. After the separation with 8% SDS-PAGE, the membranes were transferred onto polyvinylidene difluoride (PVDF) membranes. Subsequently, the membranes were blocked with 5% non-fat milk and then incubated with primary antibodies specific to connective tissue growth factor (CTGF; cat. no. ab209780; 1:1000; Abcam), Fibronectin (cat. no. ab268020; 1:1000; Abcam), alpha-smooth muscle actin (α-SMA; cat. no. 14395–1-AP; 1:1000; Proteintech), KLF5 (cat. no. ab137676; 1:1000; Abcam), phosphorylated (p)-p38 (cat. no. ab178867; 1:1000; Abcam), p-c-Jun N-terminal kinase (p-JNK; cat. no. ab307802; 1:1000; Abcam), p38 (cat. no. ab170099; 1:1000; Abcam), JNK (cat. no. ab199380; 1:2500; Abcam), or GAPDH (cat. no. ab9485; 1:2500; Abcam) at 4 °C overnight, following which was the incubation with horseradish peroxidase (HRP)-labeled goat anti-rabbit secondary antibody (cat. no. ab6759; 1:5000; Abcam) at room temperature for 2 h. Finally, the protein bands were visualized with enhanced chemiluminescence (ECL) Detection Reagent (Yeasen Biotech) and analyzed with Image J software (Version 1.8.0).

### Immunofluorescence assay

Following the indicated treatment with AngII and MOR or the transfection with oe-KLF5, AngII-induced HCF cells were fixed with 4% paraformaldehyde at 4 °C for 15 min and permeabilized with 0.2% Triton X-100 at 37 °C for 30 min. After the block with 10% bovine serum albumin (Thermo Fisher Scientific, Inc.), AngII-induced HCF cells were incubated with primary antibodies specific to Collagen I (cat. no. ab138492; 1:1000; Abcam) and Collagen III (cat. no. ab184993; 1:100; Abcam) at 4 °C overnight. On the next day, the cells were incubated with 100 µl/well working solution containing Alexa Fluor 488-conjugated goat anti-rabbit secondary antibodies (cat. no. ab150077; 1:200; Abcam) at room temperature for 1 h. 4'-6-diamidino-2-phenylindole (DAPI) was employed for nuclear counterstaining, and an inverted fluorescence microscope (Olympus Corporation) was used for observation.

### Reverse transcription-quantitative PCR (RT-qPCR)

Total RNA extracted from sample HCF cells using Trlzol® reagent (Biosharp) was reverse transcribed into complementary DNA (cDNA) with QuantiTect Reverse Transcription kit (Qiagen GmbH) according to the manufacturer’s instructions. The cDNA templates were amplified by means of SYBR Green PCR Master Mix (Takara, Toyobo, Japan) on the 7500 Fast Real-time PCR system (ABI, USA) according to the manufacturer’s instructions. Finally, the relative gene expression was calculated with the 2^−△△CT^ method (Livak and Schmittgen 2001). The following were the sequences of primers: KLF5 forward primer: 5′-CGCTTGGCCTATAACTTGGTTC-3′, reverse primer: 5′-GGTCTACGACTGAGGCACTG-3′ or GAPDH forward primer: 5′-TGTGGGCATCAATGGATTTGG-3′, reverse primer: 5′-ACACCATGTATTCCGGGTCAAT-3.

### Statistical analysis

All experimental data were displayed in the format of mean ± standard deviation (SD) and analyzed with GraphPad Prism 8.0 software (GraphPad software, Inc.). The comparisons among multiple groups were demonstrated by virtue of one-way analysis of variance (ANOVA) with Tukey’s post hoc test. *P* less than 0.05 meant that all experimental data were of statistical significance.

## Results

### MOR inhibited the hyperproliferation of AngII-induced HCF cells

**Fig. 1:**
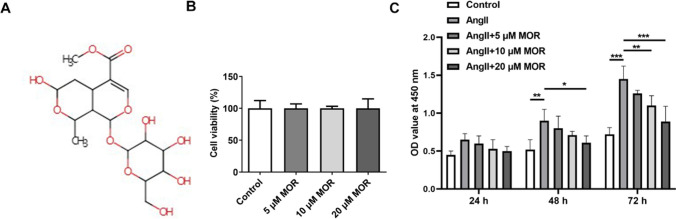
MOR inhibited the hyperproliferation of AngII-induced HCF cells. **A** The chemical structure of MOR. **B** The viability of MOR-treated HCF cells was detected using CCK-8. **C** The proliferation of AngII-induced HCF cells with MOR treatment was detected using CCK-8. *P < 0.05, **P < 0.01, and ***P < 0.001

The chemical structure of MOR is presented in Fig. 1A. After the treatment with different concentrations of MOR (5, 10, and 20 µM), cell viability was assessed with CCK-8, and the results showed that MOR had no significant effects on the viability of HCF cells relative to the Control group (Fig. 1B). Compared with the Control group, AngII induction significantly enhanced the proliferation of HCF cells. By contrast with the AngII group, MOR treatment concentration-dependently reduced the proliferation of AngII-induced HCF cells (Fig. 1C).

### MOR inhibited the migration of AngII-induced HCF cells

**Fig. 2:**
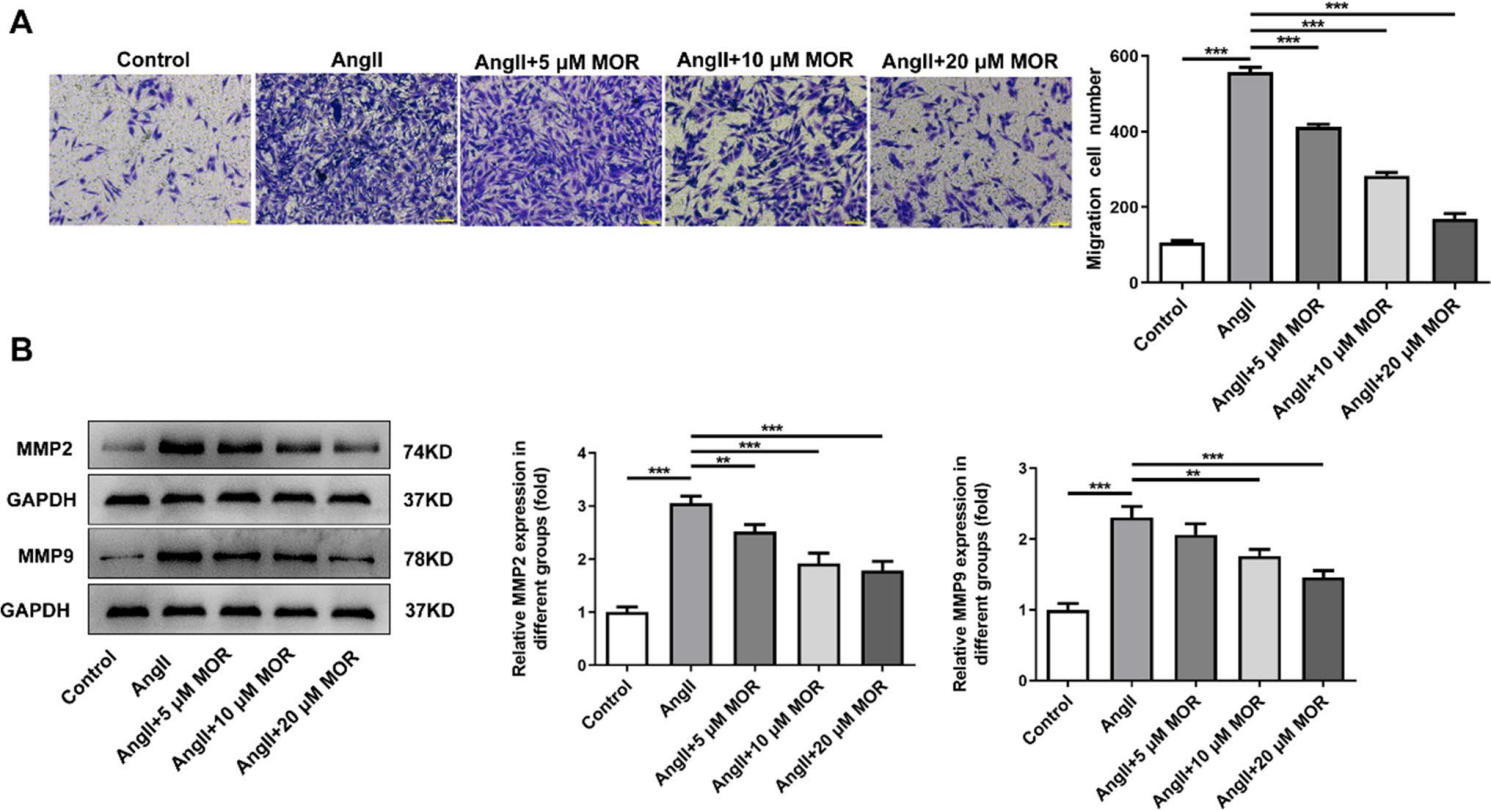
MOR inhibited the migration of AngII-induced HCF cells. **A** The migration of AngII-induced HCF cells with MOR treatment was detected using transwell assay. **B** The activities of MMP2 and MMP9 in AngII-induced HCF cells with MOR treatment were detected using gelatin zymography analysis . **P < 0.01 and ***P < 0.001

Results obtained from transwell assay demonstrated that AngII induction conspicuously increased the migration of HCF cells when compared with the Control group, which was then inhibited by MOR treatment (Fig. 2A). Results obtained from gelatin zymography analysis revealed that the increased activities of MMP2 and MMP9 in HCF cells due to AngII induction were greatly reduced following the treatment of MOR in comparison with those in AngII group (Fig. 2B).

### MOR inhibited the fibrosis of AngII-induced HCF cells

**Fig. 3:**
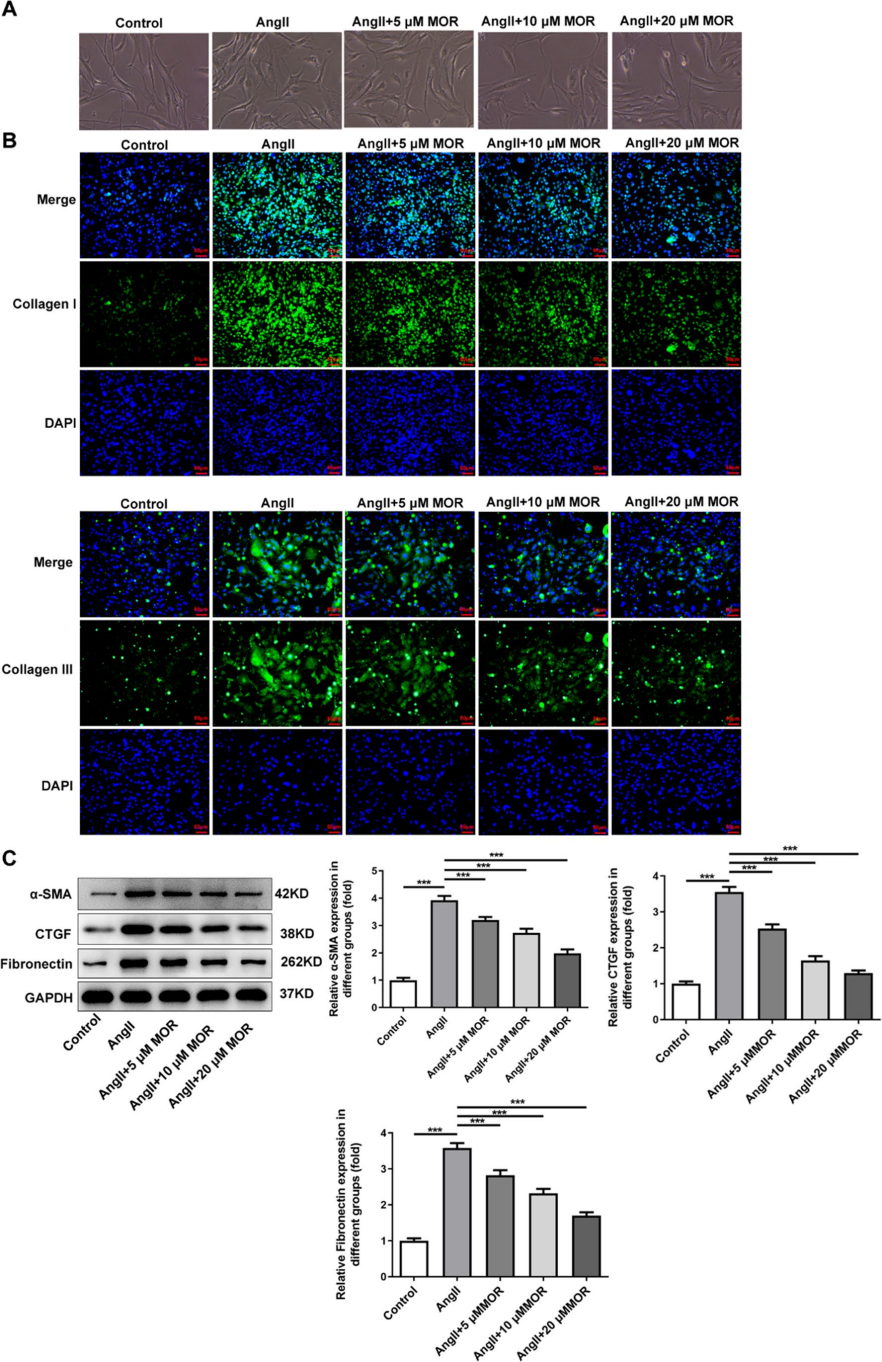
MOR inhibited the fibrosis of AngII-induced HCF cells. **A** The cell morphology. **B** The expressions of Collagen I and Collagen III in AngII-induced HCF cells with MOR treatment were detected using an immunofluorescence assay. **C** The expressions of fibrosis-related proteins in AngII-induced HCF cells with MOR treatment were detected using western blot. ***P < 0.001

As Fig. 3A depicted, cell size was increased, and cell morphology was flat after AngII induction, which was then partially improved following MOR treatment. Results from the immunofluorescence assay demonstrated that AngII induction markedly increased the expressions of Collagen I and Collagen III in HCF cells compared with the Control group, which were subsequently declined by MOR treatment (Fig. 3B). By contrast with the AngII group, the increased expressions of CTGF, Fibronectin, and α-SMA in HCF cells because of AngII induction were reduced after MOR treatment (Fig. 3C).

### MOR downregulated the expression of KLF5 in AngII-induced HCF cells to block p38/JNK signal pathway

**Fig. 4:**
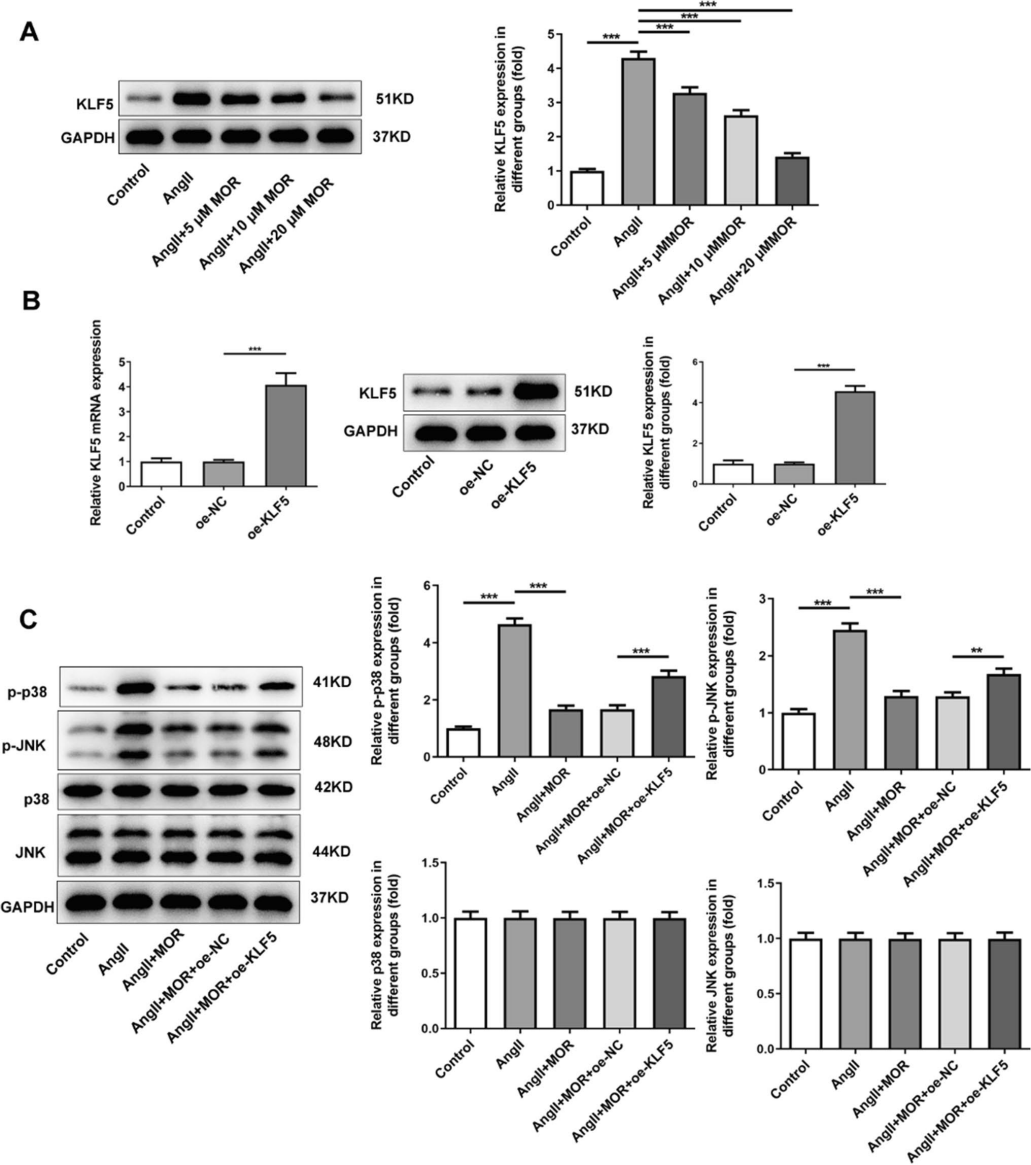
MOR downregulated the expression of KLF5 in AngII-induced HCF cells to block p38/JNK signal pathway. **A** The expression of KLF5 in AngII-induced HCF cells with MOR treatment was detected using western blot. **B** The transfection efficacy of oe-KLF5 was detected using RT-qPCR and western blot. **C** The expressions of p38/JNK signal pathway-related proteins in AngII-induced HCF cells with MOR treatment were detected using western blot. **P < 0.01 and ***P < 0.001

Compared with the AngII group, MOR treatment reduced the expression of KLF5 in AngII-induced HCF cells in a concentration-dependent manner (Fig. 4A). It was noted that 20 µM MOR contributed lower expression of KLF5 in AngII-induced HCF cells; in this way, 20 µM MOR was chosen for subsequent experiments. To upregulate KLF5 expression, oe-KLF5 was transfected into cells and RT-qPCR as well as western blot was applied to examine transfection efficacy. As Fig. 4B demonstrated, the mRNA and protein expressions of KLF5 were significantly increased by oe-KLF5 in comparison with those in oe-NC group. By contrast with the Control group, AngII induction greatly elevated the expressions of p-p38 and p-JNK in HCF cells, which were then reduced by MOR treatment (Fig. 4C). Compared with the AngII + MOR + oe-NC group, KLF5 overexpression partially increased the expressions of p-p38 and p-JNK in AngII-induced HCF cells with MOR treatment.

### MOR inhibited the proliferation and migration of AngII-induced HCF cells through the downregulation of KLF5

**Fig. 5:**
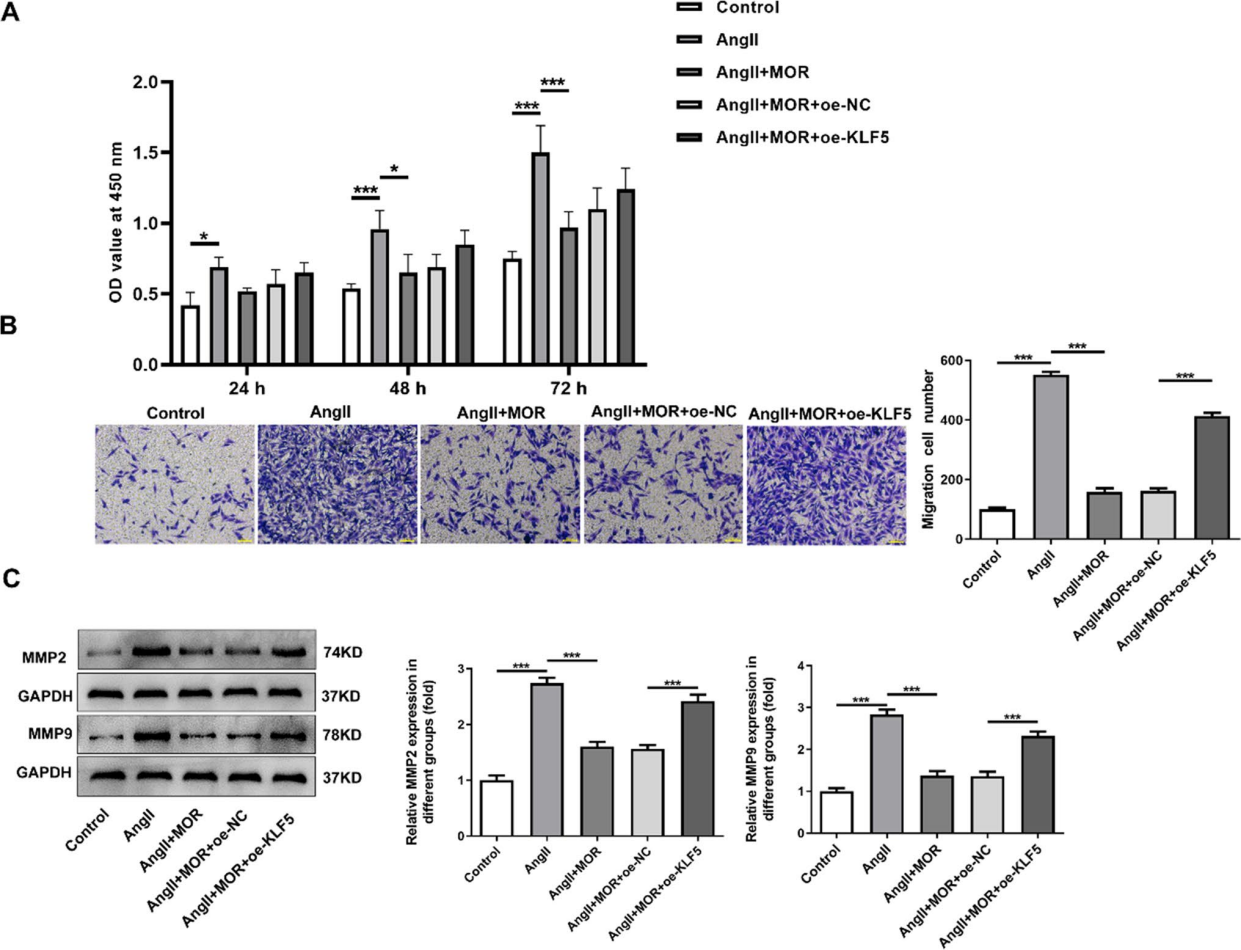
MOR inhibited the proliferation and migration of AngII-induced HCF cells through the downregulation of KLF5. **A** The proliferation of transfected AngII-induced HCF cells with MOR treatment was detected using CCK-8 assay. **B** The migration of transfected AngII-induced HCF cells with MOR treatment was detected using transwell assay. **C** The activities of MMP2 and MMP9 in transfected AngII-induced HCF cells with MOR treatment were detected using gelatin zymography analysis. *P < 0.05 and ***P < 0.001

Relative to the AngII group, MOR treatment decreased the proliferation of AngII-induced HCF cells, which was subsequently revived after overexpressing KLF5 expression (Fig. 5A). Similarly, the decreased migration in AngII-induced HCF cells with MOR treatment was increased by KLF5 overexpression (Fig. 5B). Besides, MOR treatment was discovered to reduce the activities of MMP2 and MMP9 in AngII-induced HCF cells when compared with those in AngII group, which were then elevated following the transfection with oe-KLF5 (Fig. 5C).

### MOR inhibited the fibrosis of AngII-induced HCF cells through the downregulation of KLF5

**Fig. 6:**
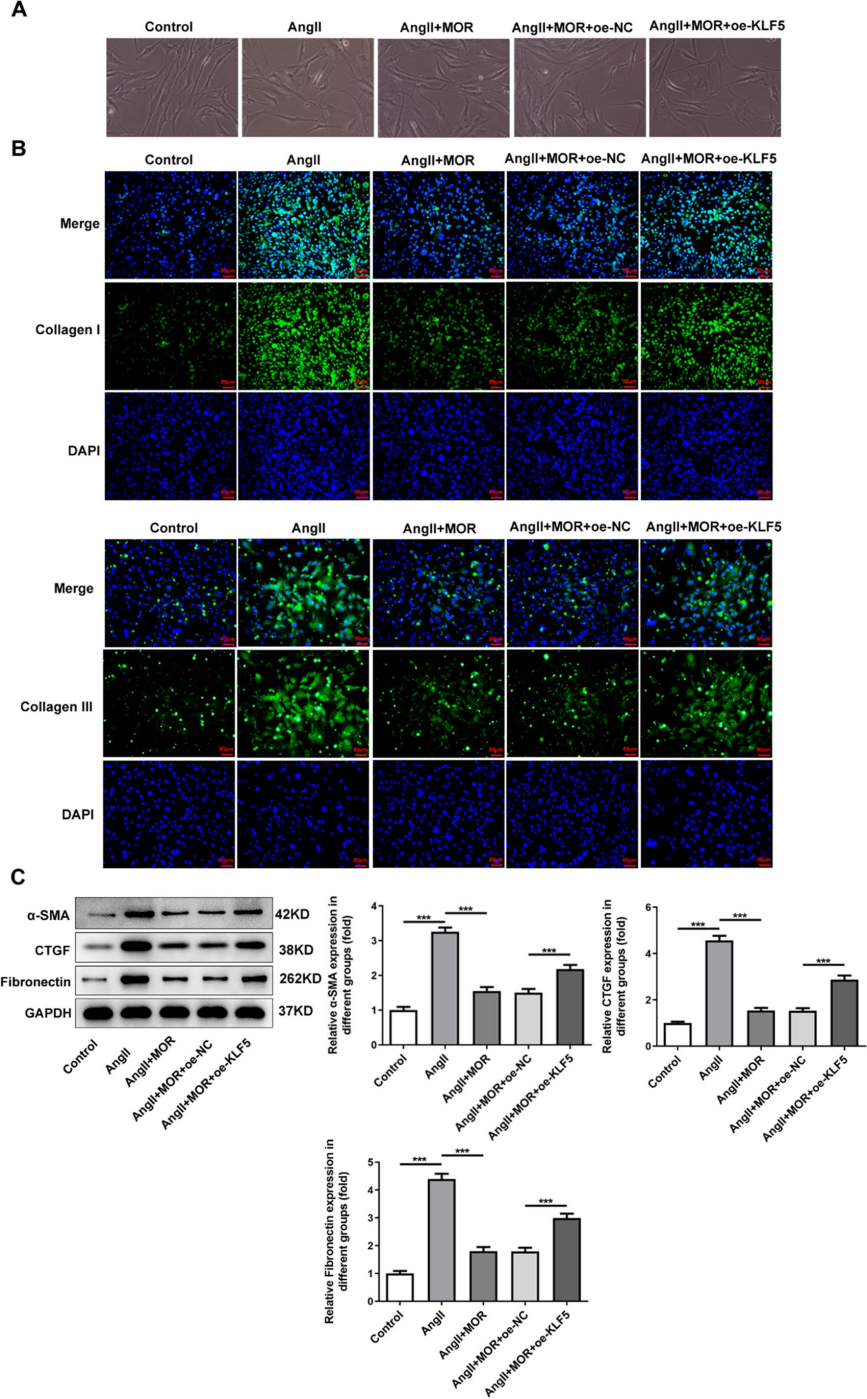
MOR inhibited the fibrosis of AngII-induced HCF cells through the downregulation of KLF5. **A** The cell morphology. **B** The expressions of Collagen I and Collagen III in transfected AngII-induced HCF cells with MOR treatment were detected using immunofluorescence assay. **C** The expressions of fibrosis-related proteins in transfected AngII-induced HCF cells with MOR treatment were detected using western blot. ***P < 0.001

As Fig. 6A displayed, the decreased cell size in the AngII + MOR group was increased by KLF5 overexpression. Relative to the Control group, AngII induction remarkably increased the expressions of Collagen I and Collagen III in HCF cells, which were subsequently reduced by MOR treatment. Compared with the AngII + MOR + oe-NC group, the expressions of Collagen I and Collagen III in AngII + MOR + oe-KLF5 were elevated (Fig. 6B). In addition, by contrast with the AngII group, MOR treatment reduced the expressions of CTGF, Fibronectin, and α-SMA in AngII-induced HCF cells, while KLF5 overexpression exhibited opposite effects on these proteins, evidenced by increased expressions of CTGF, Fibronectin, and α-SMA in AngII + MOR + oe-KLF5 group (Fig. 6B).

## Discussion

As a common pathological manifestation of many CVDs, MF can disrupt the myocardial structure, resulting in myocardial disarray as well as vasomotor dysfunction (Gonzalez et al. 2018). AngII, which is a central signaling molecule of the renin-angiotensin system, serves as a critical player in MF (Shu et al. 2021). Besides, not only does AngII induce cardiomyocyte hypertrophy but also stimulate the proliferation of cardiac fibroblasts and collagen synthesis, thereby promoting the occurrence of MF (Wang et al. 2014). In view of this, 100 nM AngII was applied to induce fibrosis in HCF cells. Then, the effects of AngII induction on cell viability were detected by CCK-8, and the results showed that AngII stimulation greatly enhanced the viability of HCF cells, which was consistent with the results in a previous study (Chen et al. 2021).

Being a main active component of iridoid glycosides from *Cornus officinalis*, MOR has been reported to have anti-fibrosis and cardiovascular protection properties (An et al. 2022; Gao et al. 2021). Current studies have evidenced that MOR suppresses fibrosis in many organs. Take pulmonary fibrosis as an example, MOR reduces the expressions of inflammatory cytokines in LPS-induced RAW264.7 and inhibits the fibrosis marker in pulmonary fibrosis mice (Chen et al. 2022). In addition, You-gui Pill (YGP) conspicuously decreases unilateral ureteral obstruction (UUO)-induced inflammatory cell infiltration, tubular atrophy, and interstitial fibrosis, thus ameliorating renal tubulointerstitial fibrosis (Wang et al. 2015). It is worth mentioning that MOR is one of the active components of YGP. In the present study, it was found that MOR inhibited AngII-induced HCF cell proliferation, migration, and fibrosis.

It is acknowledged that one of the typical manifestations of MF is the proliferation of cardiac muscle fibroblasts, and the inhibition of cell proliferation has evidenced to be effective for the amelioration of MF (Li et al. 2021; Tan et al. 2021). Many studies have found that MOR can regulate cell proliferation. For instance, Hu and co-workers have evidenced that MOR promotes the proliferation in rat mesenchymal stem cells (Hu et al. 2013). Additionally, it has also found that MOR treatment inhibits the proliferation of advanced glycation end product-induced renal mesangial cells (Xu et al. 2006). In this study, it was found that MOR with different concentrations (5, 10, and 20 µM) had no toxic effect on cells. Nevertheless, the enhanced proliferation of HCF cells induced by AngII was decreased by MOR treatment in a concentration-dependent manner.

As is known to all, migration plays an important role in MF, and the promotion of migration in AngII-induced cardiac fibroblasts can facilitate the development of MF (Pan et al. 2018). A case of previous study has evidenced that MOR treatment can increase cell migration in hair loss (Zhou et al. 2018). Liu et al. have put forward that MOR treatment can promote the migration in rat coronary artery endothelial cells (Liu et al. 2022). Here, the increased migration of HCF cells induced by AngII was subsequently inhibited following the treatment of MOR. Besides, it has been reported that MMPs can regulate endothelial cell migration, and the increase in MMP2 and MMP9 has been validated to promote cell migration (Zhang et al. 2017b; Karagiannis and Popel 2006). Moreover, Siddesha et al. have illuminated that AngII-induced cardiac fibroblast migration is mediated by MMP2 and MMP9 (Siddesha et al. 2013). In this study, AngII induction increased the protein expressions of MMP2 and MMP9 in HCF cells, which were then reduced by MOR treatment.

MF, which involves remodeling of the extracellular matrix (ECM), is characterized by excessive deposition and abnormal distribution of collagen (Kong et al. 2014). The direct targeting of collagen is supposed to be a promising strategy for the improvement of MF (Wan et al. 2019). A previous study has elucidated that MOR treatment can reduce the expression of fibrosis-related marker Collagen I in mice with pulmonary fibrosis (Chen et al. 2022). In our experiments, the increased expressions of Collagen I and Collagen III in HCF cells because of AngII induction were reduced by MOR treatment, indicating the inhibitory effects of MOR on cell fibrosis in MF. Gao and co-workers have claimed that CTGF is a potent profibrotic factor implicated in the AngII-induced pathologic fibrosis process (Gao et al. 2007). Here, AngII induction greatly elevated the expressions of CTGF, Fibronectin, and α-SMA in HCF cells, which were subsequently reduced by MOR treatment.

KLF5 was testified to control fibrosis in the heart, lungs, liver, skin, and other organs (Noda et al. 2014). Take tubulointerstitial fibrosis as an example, KLF5 expression is upregulated in MK-8617-induced HK-2 cells (Li et al. 2019). Besides, the Super-PRED database predicted that MOR can target and regulate KLF5. In this study, KLF5 expression was conspicuously increased in AngII-induced HCF cells, which was then reduced by MOR treatment. Previous study has elaborated that the block of JNK pathway by conophylline can inhibit AngII-induced MF (Zhang et al. 2021). It has also been testified that the stimulated p38 MAPK by miR-33 facilitates the development of MF (Chen et al. 2018). Evidently, JNK pathway and p38 play an important role in MF. Interestingly, the downregulation of KLF5 has been testified to block JNK signal pathway (Chang et al. 2020). In this study, it was discovered that MOR treatment decreased the expressions of p-p38 and p-JNK in AngII-induced HCF cells, which were then increased after overexpressing KLF5, indicating that MOR blocked p38/JNK signal pathway in AngII-induced HCF cells via downregulating KLF5 expression. Moreover, previous studies have demonstrated that p38/JNK is involved in MF, and the inhibition of p38/JNK can inhibit cell proliferation, migration, and α-SMA expression in AngII-induced cardiac fibroblasts (Song and Ren 2019; Liu et al. 2023). Our further experiments showed that MOR treatment inhibited cell proliferation, cell migration, and cell fibrosis in AngII-induced HCF cells by blocking p38/JNK signaling pathway through the downregulation of KLF5.

## Conclusion

To sum up, this study investigated the impacts of MOR on cell proliferation, cell migration, and cell fibrosis in MF and identified that MOR blocked p38/JNK signaling pathway via the downregulation of KLF5, which for the first time revealed the mechanism by which MOR protected against MF.

## Limitation of study

Our study also has some limitations. For example, this study preliminarily explored the regulatory role of MOR in KLF5, while the detailed mechanism has not been investigated, which will be the research focus of our future studies.

## Data Availability

No datasets were generated or analysed during the current study.
